# Book Reviews

**Published:** 1822-01

**Authors:** 


### CRITICAL ANALYSIS
### OF
### ENGLISH AND FOREIGN LITERATURE
### RELATIVE TO THE VARIOUS BRANCHES OF
### Medical Science.

Quæ laudanda forent, et quæ culpanda, vicissim

Illa, prius, cretâ; mox hæc, carbone, *notamus*.—PERSIUS.

### DIVISION I.

### ENGLISH.

Art. I.—*Observations on those Diseases of Females which are attended by Discharges. Illustrated by Copper-plates of the Diseases.* By Charles Mansfield Clarke, M.R.C.S. Lond. Part II. Royal 8vo. pp. vi. 243; four plates. Longman and Co. London, 1821.

WE cannot help feeling a degree of embarrassment in taking upon ourselves to review the continuation of a work, respecting which our predecessors, on the publication of the first part, expressed their unqualified approbation; lest we should chance to find good cause, during the attentive perusal of the present volume, for differing from them in opinion, and yet be tempted to suppress our real sentiments, from a desire to avoid the appearance of contradiction.

We are aware that there are those, who think that the opinion of an old editor of a critical journal ought to be binding on a new one; but such a doctrine is neither warranted by reason, nor by the laws of impartiality; and, were we, therefore, to find good reason to doubt the correctness of a previous opinion proclaimed in this Journal, we should feel ourselves at liberty to express our dissent from it, without the least hesitation.

On the other hand, we are afraid that, should we see occasion for repeating the eulogiums which have already been passed on Mr. Clarke, in one of the former volumes of our Journal; some of our readers might attribute this perpetual strain of praise, on our part, either to predilection, private feelings, or the desire of courting a man in high favour and practice.

To say that we neither fear the first, nor deserve the latter, of these imputations, is to advance that for which we shall doubtlessly find credit among many of our readers. Still there is something, in both terms of the alternative, which, as we observed before, places us in a situation of some embarrassment, on assuming the office of critic on the present occasion.

We will, however, enter cheerfully on the discharge of our duty; and "evil be to him who evil thinks."

There are three points on which we are compelled, *in limine*, to find fault with our author. First, with the title of his book; secondly, with his classification of female diseases; thirdly, with his introductory remark, that the "diseases affecting the female organs of generation were not generally known to practitioners," (before he offered to them the former part of his work.) Our readers will admit that these are but trifling deficiences; and that, if in them alone consist the motives for detracting from Mr. Clarke's performance, it will be no difficult matter for us to prove, ere we have done with it, that the volume before us is a work of considerable merit.

We find fault with the title of Mr. Clarke's book:

1. Because the idea, immediately suggested by it, is not of the most pleasing description; whereas, in treating of female complaints, all such ideas or associations should be avoided; and, in the present case, this might have been easily accomplished.

2. Because the *female* diseases attended by discharges are *not* the only diseases treated of in the body of the work; where we find, also, the consideration of diseases common to both sexes, often *unattended* by discharges,—such as the hemorrhoids, the presence of ascarides in the rectum, and the carcinoma recti.

We find fault with Mr. Clarke's classification of female diseases:

1. Because it is not strictly founded on facts; as many of the diseases, which he has characterized by *transparent* mucous discharge, will, at some period or other, subsequent to their manifestation, be found attended by a *white* mucous discharge: as in the case in procidentia uteri, and carcinoma of the same organ.

2. Because both the transparent and the white mucous discharge are occasionally met with at one and the same time,—as in metritis, for instance.

3. Because the white mucous discharge, being only a modification of the transparent mucous discharge, cannot be assumed as a distinctive character of a specific class of diseases.

4. Because, what Mr. Clarke has called watery discharge is, in fact, nothing but a transparent mucous discharge, somewhat diluted; as animal chemistry has proved.

5. Because, to assume (as Mr. Clarke affirms that it is possible to do,) that the knowledge of the nature of the discharge present in any female disease will enable the practitioner to solve the question as to the precise nature of the disease itself,* is to assume what few experienced practitioners will admit; particularly those who have ample and frequent opportunities of

---

* See Preface to Part I.

examining female diseases, in Dispensaries of extensive practice.*

We find fault with Mr. Clarke's introductory remarks, "that the diseases affecting the female organs of generation were not generally known to practitioners" before he offered to them the former part of his work :

1. Because, previously to the year 1814, at which epoch Mr. Clarke's first volume made its appearance, we possessed several *ex professo* works, by eminent British practitioners, on diseases of women, in which those of the sexual organs were particularly included.†

2. Because, on the continent, and more especially in France, works specifically written on diseases of the parts of re-production in females were both numerous and voluminous; and, in one or two of them, indeed, something like Mr. Clarke's classification had already been attempted.‡

Having thus propounded our reasons for venturing to differ, *ab ovo*, from our author; reasons which our readers will do well to balance in their mind, ere they decide whether we are right or wrong in what we have advanced ; we shall proceed to the analysis of the contents of the book itself, for which, we dare say, our readers are, by this time, become greatly impatient.

The volume is divided into three chapters, each of which treats of one particular discharge, and is subdivided into as

---

* A female applies to a practitioner who has adopted the classification of sexual diseases on which we are commenting, and desires to be made acquainted with the nature of a particular complaint affecting the organs of re-production, under which she labours. After a *proper* investigation of the case, which leads the practitioner to believe that there is *something* wrong in the parts, he examines the discharge adhering to his fingers, and thereby acquires the *certitude* that the disease is not an incipient polypus of the uterus, nor a carcinoma uteri, (because the discharge is not transparent and mucous,) but an inflammation of the cervix uteri, or of the substance of the uterus; these diseases being said to be attended by a white mucous discharge, which the practitioner has, in fact, met with in his examination. We now ask gentlemen versed in these matters, whether the practitioner in question is likely to be correct in his diagnosis, formed on such grounds? We answer, No! Yet the author's observations, in the work before us, seem, precisely, intended to teach us that some such facility of diagnosis, as that just mentioned, may be acquired by adopting his classification.—Is it not somewhat singular that, neither under the head of white mucous discharge, nor under that of purulent discharge, the subject of gonorrhœa in females is to be met with?

† MANNING, *Treatise on Female Diseases.*—LEAKE, *Medical Instructions towards the Prevention and Cure of chronic or slow Diseases peculiar to Women.*—HAMILTON, *a Treatise on the Management of Female Complaints, &c.* besides the many observations in Burns, Denman, Johnson, and others.

‡ CHAMBON, *Maladies des Femmes*, 8 vol. 1800 —VIGAROUS, *Cours Elementaire des Maladies des Femmes*, 2 vol. 1801.—PLATIN, *du Catarrhe Uterin*, 1803. In this work a great stress is laid on the mode of distinguishing diseases of the organs of generation in women, according to the nature of the discharge, as well as from the chemical difference existing, between one discharge, and another.—GARDIEN, in his *Traité d'Accouchemens*, 1807.—CAPURON, *Traité des Maladies des Femmes*, 8vo. 1812.—Since which period, two other very valuable works have been published, by Dr. NAUCHE and Dr. PATRIX, on utero-vaginal diseases many sections as there are diseases attended by the discharge first mentioned at the head of the chapter.

Mr. Clarke admits five species of discharge:—" 1. Transparent mucous discharge. 2. White mucous discharge. 3. Watery discharge. 4. Purulent discharge. 5. Sanguineous discharge. But he has omitted to inform us on what solid chemical distinction this arrangement it founded.

1. The morbid states of the sexual organs in females which are attended by the first of the above-mentioned discharges, formed the subject of the first part of the author's work published in 1814.

2. Those which are characterized or accompanied by a *white mucous* discharge are—

*a.* Inflammation of the cervix uteri.
*b.* Inflammation of the substance of the unimpregnated uterus.

3. The sexual diseases in which we meet with a *watery* discharge are said to be—

*a.* Cauliflower excrescence.
*b.* Hydatids of the uterus.
*c.* Oozing tumour of the labium.

4. The *white purulent* discharge is found to accompany—

*a.* Inflammation of the mucous membrane of the uterus.
*b.* Inflammation of the mucous membrane of the vagina.
*c.* Ulceration of the os uteri and cervix uteri.
*d.* Corroding ulcer of the os uteri.
*e.* Ulcerated carcinoma of the rectum.
*f.* Ulcerated carcinoma of the uterus.

5. Of the diseases attended by *sanguineous* discharge, no mention is made.

*White Mucous Discharge.*—Its characters, according to Mr. Clarke, are as follow: " This discharge is opaque, of a perfectly white colour; and it resembles, in consistence, a mixture of starch and water made without heat, or thin cream; it is easily washed from the finger after an examination; and it is capable of being diffused through water, rendering it turbid." In many instances, the white mucous discharge is much thicker than cream, having the tenacity of glue; and, perhaps, this is the state in which it comes away from the cervix uteri. This discharge is never met with in large quantity; and how far it may be the forerunner of disease in the cervix of the uterus, the author is not prepared to say.

*Watery Discharge.*—By this term, Mr. Clarke means " that form of discharge which resembles clear water, having no colour, and which contains very little glutinous matter, sometimes none at all."

*Purulent Discharge.*—" The characters of this discharge are, a heavy, yellowish, opaque fluid, possessing little tenacity."

The quantity of it is seldom very profuse, and never so considerable as that of the watery discharge; yet it is sometimes the cause of so much debility, that the powers of the constitution are quickly expended.

INFLAMMATION OF THE CERVIX UTERI.—"Where an examination *per vaginam* has been made, the external parts and the canal of the vagina have not possessed a more than ordinary degree of sensibility; but, upon the finger reaching the cervix uteri, the patient has complained of pain, and the uneasiness has been compared to that which has been experienced upon the passage of an evacuation from the rectum; pressure, in both cases, being the cause of the pain. There is, however, no alteration of structure in the part: no thickening, no peculiar enlargement of the os uteri, no breach of surface; the portion of the vagina which is reflected over the cervix uteri possessing its usual polished and smooth state."

Several cases occurred to the author, attended by the symptoms above mentioned, before he ascertained the nature of the disease; but he now asserts, without hesitation, that, wherever the white mucous discharge is present, there will be found, on examination, a tenderness of the cervix of the uterus. This complaint is generally relieved by an appropriate treatment, of which the following case will give a very satisfactory description:

"A lady, about twenty-five years of age, who had been married two or three years, but who had never fallen with child, complained of a considerable degree of uneasiness at the extremity of the back, near the os coccygis: on this account, she indulged much in the horizontal posture. She had also been liable, for some years, to a discharge from the vagina, which, on investigation, was ascertained to put on a white appearance. The general health was tolerably good, excepting that, at the periods of menstruation, great pain was felt at the bottom of the belly, which lasted for twenty-four hours; during which time the menstruous discharge did not flow freely, but was pale, and occasionally mixed with portions of a stringy substance. On account of the discharge, astringent injections had been employed by a practitioner, who had been consulted; but without any effect upon the complaint or its symptoms. Tonics had also been exhibited, without any advantage. An examination being allowed, the uterus was found unusually low, and the neck of it possessed a much greater degree of sensibility than is common; so that, pressure being made upon it, the patient complained much: but this increased sensibility did not extend to the neighbouring parts; neither was there any alteration in the structure of the parts. This lady had been in the habit of taking much riding exercise, and it is more than probable that to this cause was to be attributed both the tenderness of the cervix uteri and the descent of the whole organ. Prolapsus uteri being a very infrequent disease in women who have not borne children, the patient was desired to lose several ounces of blood from the loins, to live temperately, to avoid riding exercise, to take only a sufficient quantity of walking exercise to keep herself in health, and to inject some tepid water into the vagina. Sexual intercourse was, of course, interdicted. Soon after this plan was instituted, the symptoms diminished. On account of the painful menstruation, some diaphoretic medicines, with opium, and the use of the hip-bath, were recommended; and the sufferings at the periods were subdued. At the end of three or four months, the complaints were removed; but the patient did not become pregnant."

The pain in the back, and at the lowest part of the abdomen, is sometimes very violent in this complaint; and occasionally the bladder is so much affected, that the author relates a case in which the use of the catheter was required on several occasions. Generally speaking, the constitution does not sympathize with the local irritation; but cases of exception to this rule are occasionally met with.

*Treatment.*—"In ordinary cases, the most successful mode of treatment is to take away some blood, either by cupping or by leeches applied to the groins or to the back; and it may be necessary to repeat the local blood-letting several times. If symptomatic fever should be present, it will be prudent to open a large vessel; but this is seldom requisite, and all useful purposes are answered by local blood-letting. The hip-bath proves a useful remedy, and the patient may sit in it twice a-day, the water being heated about ninety degrees. Where the hip-bath cannot be conveniently procured, fomentations of warm water, or of decoctum papaveris, to the back or to the abdomen, will be found serviceable; and, even during their application, the patient is frequently made much more comfortable. Tepid water, thrown into the vagina with a syringe, constitutes a direct fomentation to the part affected, and it may be recommended with advantage. It is very useful to keep the bowels in a relaxed state; and, for this purpose, small doses of sulphate of magnesia will answer exceedingly well in plethoric habits, and it may be exhibited in any agreeable vehicle. In languid habits, it may be advisable to substitute expressed oil in its stead. Half an ounce of ol. ricini may be given in the morning, or two drachms in the morning and at noon, with a drachm of manna. All the purgatives which irritate the lower part of the bowel should be carefully avoided."

Inflammation of the Substance of the unimpregnated Uterus.—This disease, or metritis, is not unfrequent. In the violence of the pain which attends it, there appears to be a great difference, observes the author, between the inflammation of the womb in its impregnated state, or soon after delivery, and in its usual unimpregnated state. We profess not to comprehend the whole force of the explanation of this fact, offered by Mr. Clarke, which, after all, is a very natural one. It is here stated, that the *nerves* of the uterus become larger in pregnancy: has Mr. Clarke ascertained the correctness of this observation by actual demonstration? for we confess to have seen some few specimens of the uterus immediately after parturition followed by death, where, on dissection, the nerves were found not to be altered in size.

*Symptomatology.*—Metritis is accompanied by "a constant uneasiness referred to the pelvis; and this gradually increases, seldom becoming intensely violent, but greatly interfering with the comfort of the patient, who complains of pain, sometimes at the bottom of the abdomen, and sometimes in the back. Superadded to the permanent pain, violent pains occur by paroxysms, with irregular intervals: these are of short duration."

One of the distinguishing symptoms of acute metritis, which the author has not mentioned, is the direction which the pain seems to take, during the most violent paroxysms of the complaint, beginning in the womb, and shooting obliquely downwards and outwards, as far as the tuberosity of the ischium; where, indeed, the slightest pressure, on such occasions, produces the most exquisite uneasiness.

*Treatment.*—Mr. Clarke prefers topical to general blood-letting, by means of cupping on the sacrum, or the application of leeches to each groin. Fomentations, consisting of a mixture of tincture of opium, and a decoction of chamomile, should be used twice or thrice in the twenty-four hours; and, where the symptoms are not disposed to yield, the patient should be kept in bed, and small doses of antimony given in a saline draught, with three or four drops of tincture of opium, or one drachm of syrup of poppies. Purging should, by all means, be employed. The diet of the patient should be light, and not stimulating.

We recommend to the earnest attention of our readers Mr. Clarke's observations on amenorrhœa, with which the first chapter concludes. They will be found full of practical applications; such, indeed, as one had a right to expect from a practitioner of so much experience.

CAULIFLOWER EXCRESCENCE OF THE OS UTERI.—This is the first of the diseases mentioned by the author under the head of *watery* discharge. The surface of the tumor is granulated, and consists of a great number of small projections, which may be picked from the surface. A very fine membrane is spread over it, and from this membrane is poured out that aqueous secretion which characterizes, in a marked manner, this disease. The tumor occupies the upper part of the vagina; but it will also be found, at times, to fill the whole canal, and to protrude between the labia. Its colour is a bright red. If the membrane covering the tumor has been injured in an examination, the blood-vessels immediately beneath it pour out their contents, which appear to be florid red blood. Mr. Clarke asserts, that the attachment of the cauliflower excrescence is to the surface of the os uteri, and to that alone. It may be generally so; but we are confident, from experience, that exceptions to this rule not unfrequently occur. The same observations may be made with regard to the similitude, in the tumor, to a cauliflower, which, sometimes, it does not bear; and again with regard to the discharge which accompanies the tumor, and which will, occasionally, be found to be either opaque or sanguinolent, or to consist of a mixture of both. A case illustrative of each of these positions has been related in an obstetrical work, published four years ago, where the practitioner had the kind assistance of Mr. Brodie; the quotation of which in this place cannot be deemed improper.

"Ancilla viginti, circiter, annos nata, formæ integræ—temperamento, ut ajunt, lymphatico, et habitu corporis mediocri, prædita; quum dolore peracuto in vagina laborasset, et frustra varia, pro levando malo, tentasset, ad me, consilium petens, accedit. Crurum ac artuum dolore conquerebatur tensivo, una cum infimi dorsi ventrisque cruciatibus. Post longos moerores et febrem in morbum inciderat suppressæ menstruæ purgationis: inde ventris intensio, spiritus difficilis, et musculorum dolor supervenerant. Parentes, de gnatâ solliciti, medicam petunt opem; et ex medicamentorum farragine (vano conamine!) salutem sperant. Morbi causâ minime notâ quis morbum curaverit? Vocatus quæstiones, circa pudendi incommoda, instituo; muliercula rubore suffusa, se laborare talibus incommodis denegat. In infimo, tamen, abdomine, levi manu contrectato, dolor, præcipue ad regionem sub-pubianam, observatur. Medicamenta, cum varia fortunâ, præscribo; et paucis sic elapsis hæbdomadis, quum omnia symptomata ingravescant atque sævire vehementer, de vera morbi indole quod suspicabar, cum matre et amicâ in colloquio communicabam. Illis suadentibus me ancilla, postremo, pudenda examinare patitur. Labia externa tumefacta erant, nymphææ vero longissime propendebant, molliores, cristatæ, nigrescentes. His sejunctis tumores quidam rubri, quasi sanguine turgescerent, et parvis cerasis non multo dissimiles, apparuere. Nec erant soli; nam digito per vaginam intromisso, alios multos ex convexitate urethræ pendentes, et vaginam pene totam obstruentes, reperii. Summum infligebant dolorem, et compressi facile mittebant sanguinem. Copiosè fluebat leucorrhœa. Me suasore ac duce instanter admittitur operatio; nec sine multis difficultatibus ligamina tumoribus externis appono. Plurimis, hoc modo, in brevi spatio dierum, dissectis, quid agerem de cæteris, qui in vagina interna extabant, diligenter animo tractabam. Erant vero isti exigui, numerosissimique, nec ligaturis se præbebant. Rebus sic stantibus, consilium peto a B. C. Brodie amico meo dilectissimo, et in officiis chirurgicis omninò expertissimo, cujus dexteritatem nec possum quin laudibus hîc extollam. Peracto examine cauterium, ex potassa impura, admovere opinatur, et ipse, semel, instituit operationem, quam egomet, bis in hæbdomada, per mensem iteravi. Evanescunt hôc methodo tumores; sed ulcus amplum, mirificè dolorosum, saniosum, sanguinolentum, quasi tota vagina una plaga esset, relinquunt; nec consolidandum nisi per aquæ cupri sulphatis injectionèm vaginalem. Hanc strenue commendavi: et sanata est. Tempus circa solstitium vernale MDCCCXVIII."*

The author has related several (nearly similar) cases in illustration of his very accurate and clear description of this disease. The symptoms are also detailed with much precision; and the treatment proposed will be found highly judicious. But we must refer to the book itself for an account of those subjects.

Hydatids of the Uterus.—" In the cavity of the uterus small vesicles, containing a limpid fluid, are sometimes met with, constituting the disease called hydatids of the uterus. These vesicles vary, considerably, in size, from that of a small currant to that of a large grape. They are connected with the uterus, and with each other, by small filaments; and portions of a substance resembling partly blood, and partly coagulated lymph, are frequently mixed with them. A similar substance is attached to the internal part of the uterus, from which the footstalks of the hydatids grow. As the number of these hydatids increases, the cavity of the uterus becomes more capacious; and when, at length, the uterus has acquired a large size, it seems to be offended by its contents, and contract upon them."*

The cause of this complaint is not ascertained.

*Symptomatology.*—Hydatids of the uterus do not appear to produce any peculiar symptoms: the pressure made by the enlarged uterus upon the surrounding parts will occasion some inconveniences,—such as retention of urine, constipation of the bowels, œdema and cramp of the lower extremities. The function of menstruation is usually interrupted. In examining a patient labouring under this complaint, the body of the uterus will be found enlarged, and suddenly bulging out from the upper part of the cervix; but the symptom which, according to the author, serves to distinguish this disease from all others and from pregnancy, is the discharge of an almost colourless watery fluid. This watery discharge is to be distinguished from that, which attends the cauliflower excrescence, by the irregularity and suddenness of its appearance and cessation, being produced by a rupture of one or more of the coats of these hydatids. In some cases, the uterus increases rapidly, in others, slowly; but in all, sooner or later, the parietes of the womb are excited to contraction.

"From this period of time, a process resembling labour commences;

---

* Report of the Practice of Midwifery at the West General Dispensary for 1818; p. 142-3.

**the os uteri is dilated; the hydatids are expelled by periodical pains; and then, for the first time, danger presents itself in the form of alarming hemorrhage. This hemorrhage is more frightful than that which follows the removal of the placenta from an uncontracted uterus: and the reason is obvious,—the placenta covered only a limited space of the internal surface of the uterus, whereas the hydatids spring from every portion of the cavity."**

*Treatment.*—The disease hitherto has not been cured artificially, nor has its progress, towards its termination, been once arrested. As symptoms arise, they are to be treated as nature requires. When the period arrives at which the uterus is striving to unload itself of its contents, then the practitioner's utmost skill and energy will be wanting, to control the hemorrhage, and sustain the powers of the constitution.

If the uterus will admit of it, Mr. Clarke advises the introduction of two or three fingers, in order to bring away the hydatids.

The strength of the patient is to be restored by nutritive diet, and the exhibition of such medicines as tend to increase the tone of the system.

Mr. Clarke mentions another variety of hydatid of the uterus, in which this viscus is distended by one single cyst, and its contents to an enormous size. This variety is a very uncommon disease, and terminates without the occurrence of any of those distressing symptoms which threaten life in the disease first described.

The oozing Tumor of the Labium.—The author has given this name to a tumor, seldom projecting far above the plane of the skin, but sometimes so large as to leave, scarcely, any part of the labium free from it. The colour of this tumor varies little from that of the cuticle in the neighbouring parts. A discharge arises from the surface of, or rather from interstices in, the tumor. The fluid which escapes is of a watery character, and is sometimes very abundant in quantity, being renewed almost immediately after the surface has been dried with a napkin. Blood never escapes from this tumor, however it may be roughly handled. The chief inconveniences of this disease are itching of the parts, sometimes a sensation of heat, and the discharge above mentioned.

*Treatment.*—When erysipelatous inflammation is present, Mr. Clarke thinks that more advantage will be derived from the internal exhibition of bark, which will, however, make no impression on the disease itself. The patient should be desired to live upon a nutritious diet, and to take a moderate quantity of wine. Much good may be obtained, in many instances, from external applications. Common starch powder; or a mixture of starch powder and sulphate of copper, finely levigated; or a solution of sulphate of copper or of nitrate of silver, may be used. A solution of gum arabic, in decoction of oak-bark, may be tried. Cold water is also a valuable remedy. Spirit, however, Mr. Clarke says, is more effectual than any other application. Brandy, arquebusade, cologne water, or even alcohol, will be found of material service. We are rather surprised that the author has not mentioned the only mode of treatment which, in our hands, has been followed with beneficial results,—namely, free incisions into the tumor, and the application of an exhausted glass. This practice, repeated for two or three days, puts an end to the disease.

Mr. Clarke has taken an opportunity, in this place, of introducing some very apt and instructive remarks on involuntary discharges of urine.

We are now arrived at the consideration of, by far, the most important part of Mr. Clarke's volume; but, as the diseases which it principally embraces will be noticed more particularly, and at full length, in another Number of our Journal, when we shall take an opportunity of reviewing the two French works on Diseases of the Womb, mentioned in a note in this article, we must reserve for that occasion our opinion on the merits of the remaining portion of the volume before us.

From all that we have thus far said of Mr. Clarke's performance, our readers must be convinced that, short of a very few blemishes, (and where is the work that has none?) the second part of his observations on certain female diseases attended by discharges, is worthy of that practitioner's high and well-merited reputation.

---

Art. II.—*Miscellaneous Works of the late* Robert Willan, m.d. f.r.s. f.a.s. *Comprising an Inquiry into the Antiquity of the Small-Pox, Measles, and Scarlet Fever, (now first published:)—Reports on the Diseases in London, (a new Edition:)—and detached Papers on Medical Subjects, collected from various Periodical Publications.* Edited by Ashby Smith, m.d. Licentiate of the Royal College of Physicians in London; Member of the Medical and Chirurgical Society of London, and of the Royal Medical Society of Edinburgh. 1 vol. 8vo. pp. xxvii. 488. T. Cadell. London, 1821.

There are three great divisions in this volume; the first of which only contains a posthumous production of the eminent practitioner mentioned in the title-page, now for the first time made public.

The two latter divisions of the volume embrace a series of papers and observations which have been long before the public; but which, being either wholly out of print, or scattered among different periodical publications, have, for some time, been difficult of access. Dr. WILLAN's Reports on the Diseases in London, contained in the middle portion of the volume, present an aggregate collection of lists of diseases prevalent in the metropolis, which, having occurred under his immediate observation, during five years of his extensive practice, private as well as public, can be relied upon for their accuracy as well as authenticity. This part of the volume abounds with valuable suggestions on diagnosis, and important practical precepts, and contains many original pathological observations. The same remark applies to the collection of papers taken from various periodical works, and now for the first time brought before the public in their present form. Though few in number, these papers will be found to retain, even at this somewhat distant period from their first publication, that value which they originally possessed, and which essentially belongs to the subjects of those papers, as well as to the able manner in which they are treated, and not to the passing fancy of fashionable caprice.

Dr. Willan's Inquiry into the Antiquity of Small-pox, Measles, and Scarlet Fever, with which the volume begins, exhibits strong evidence of that erudition and classical research for which that able physician was justly distinguished. The copious notes, and numerous references to ancient and modern authors, contained in it, must have required much patient inquiry and extensive reading. The appropriate manner in which they are brought in, and the skill with which they are in many instances appended to the original observations of the Author, are proofs of his tact and excellent judgment.

The treatise is divided into four chapters; and the purport of the whole of it seems to be to establish this position "that the small-pox, measles, and scarlet fever, have prevailed in almost every age of the world, of which history or tradition has furnished us with any records."

*In the first chapter*, Dr. Willan has endeavoured to trace vestiges of the small-pox between the third and seventh century; and, for this purpose, he has recourse to the writings of the Arabians, and particularly to those of RHAZES, who wrote an express treatise on the small-pox, and who, after a long investigation into the origin of the disease, satisfied himself that it existed in the second century.

Dr. Willan, being struck with the idea that some traces of variola might be found in the history of pestilence occurring in ancient times, instituted a further inquiry into the leading published statements on pestilence; under which denomination it is well known that the Arabians themselves often arranged many eruptive fevers; and became clearly convinced that certain parts of those statements, in almost every instance, refer to the small-pox, and to the small-pox only.

*In the second chapter*, the inquiry is continued through the writers of the first and second centuries. Philo Judæus, Herodotus, and Rufus of Ephesus, are referred to in support of the same argument; but here the proofs derived from such sources are not so positive, and can only depend upon the admission of Dr. Willan's position, that the description given by those authors of loimoid or pestilential diseases *must* refer to small-pox in many instances. These three writers have, in their descriptions, omitted the peculiarly characteristic symptoms of the proper pestilential fever, and have noticed, instead, the existence of *ulcerative*, *anthrax-like*, *herpetic exanthemata on the face and the rest of the body;* a circumstance, which Dr. Willan is disposed to consider, as conclusive of the idea that they alluded to the small-pox. Dr. Willan has also quoted a passage, in this chapter, from Dion Cassius's Roman History, affording a presumption that some mode of inoculation had been attempted in the reign of Domitian, (A. D. 92,) and revived in that of Commodus.

Much auxiliary reference of various kinds, referable to different periods of the Christian era, and in support of Dr. Willan's position, is collected in the *third chapter.*

Of the *fourth chapter*, a large part is dedicated to the purpose of controverting the position of Baron Dimsdale and others, "that the small-pox was imported from Asia at the time of the Crusades, and made its first appearance in Europe about the thirteenth century."

But here we must suffer the editor of the present volume to give an analysis of this part of the inquiry.

"Such an opinion is proved to be destitute of any foundation:—'from the frequent recurrence,' (as had been noted by M. Du Fresne,) 'in the *Acta Sanctorum*, of the terms variola, vayrola, veyrola, vayrora, variolus, and morbus varicus, between the years 800 and 1400; and from the numerous passages in MS. Chronicles and Histories, recognizing the existence of the small-pox, under its name variola, in many parts of Europe, during the ninth and tenth centuries: whereas the first crusade was not terminated till the year 1100.

"Ascending to more distant times, descriptions of pestilential eruptive diseases prevalent in France, Germany, the Netherlands, and Italy, during the sixth, seventh, and eighth centuries, under the names of pusulæ, pustulæ, pesulæ, &c. applicable to the small-pox, with different degrees of resemblance up to the most complete, are adduced from Gregory of Tours and other writers; and the existence of the last-mentioned malady in France is traced to a period long anterior to its alledged Arabian origin, by the light derived from a miscellaneous MS. in the British Museum, 'of the eighth or ninth century, partly Saxon, partly Latin; in which it is said, that St. Nicaise, bishop of

Rheims, and a martyr, A.D. 453, had been affected with a species of *variola*.'

"With respect to Britain, the completion of the design of converting the island to Christianity, under the auspices of Pope Gregory I. led to such frequent intercourse with Italy, France, and Belgium, that the epidemical and contagious disease prevailing in France at the close of the sixth century, and which has been considered to have been the small-pox, was necessarily communicated, from time to time, through the heptarchy. The establishment of religious houses contributed to the more ready propagation and extension of the disease. Instances of this kind are particularized from Bede, relating to various abbies and monasteries.

"The treatise concludes with a description, from Adomnan, a learned Hibernian Scot, of an *eruptive epidemical* disease in Ireland, attended with *purulent ulcerations*, which raged contemporaneously with the pustular lues in France, mentioned by Gregory of Tours."

In conducting his inquiry, Dr. Willan has not confined himself to the ordinary sources of information on such subjects—the writings of medical authors; he appears also to have taken a comprehensive view of the works of the historians, the poets, and the ecclesiastical writers of antiquity. From these authorities combined, he has accumulated a mass of probable evidence, which, whatever weight might be due to the parts of which it is composed, considered singly, yet, taken collectively, tends to establish the author's position. His arguments in support of that position are urged with greatest force, and supported with more direct evidence, in the first and second chapters. These, we are informed by Dr. Smith, were revised by Dr. Willan in his last illness; his farther progress in the task of correction having been terminated by death.

Of the Reports on the Diseases in London, embracing, more particularly, the consideration of those diseases which prevailed during the years 1796, 97, 98, 99, and 1800, we cannot be expected to offer any opinion. They have for many years been before the public, in a separate as well as a collected form; and we gave a general review of them in the fifth volume of our Journal.

Of the utility of such reports, from medical officers attached to extensive medical charities, we are known to entertain the strongest conviction. It is not sufficient to do good by our present exertions; we should endeavour to let posterity share in the advantages which may result from them, by transmitting to our successors a faithful record of our observations on the diseases most prevalent amongst the inferior ranks of society. Where would the science of medicine be at this moment, if physicians, zealous in the cause of humanity and intellectual advancement, had not handed down to us the results of their experience? The thanks of the profession, therefore, are due to Dr. Willan, for having set apart so much of his valuable time, in the prosecution of so laudable an object as the one he has so well accomplished; while a share of our gratitude should likewise belong to his relative and friend, Dr. Ashby Smith, who, moved by considerations purely disinterested, has undertaken to perpetuate, by his present edition, some of the productions of a physician, whose name will continue to be cherished as long as the superior class of the profession, to which he belonged, in this country, shall continue to flourish above that of every other nation.

---

### DIVISION II.

### FOREIGN.

ART. III.—*Recherches Statistiques, &c.—Statistical Inquiries relative to the City of Paris, and the Department of the Seine: with a Collection of Tables.* Drawn up by order of Count de CHABROL, Counsellor of State, Prefect of the Department. 8vo. Paris, 1821. (For private circulation only.)

STATISTICAL inquiries form one of the principal elements of civil administration. The progress which this branch of political science has made, in almost every civilized country in Europe, within the last forty years, proves the degree of estimation it has always been held in by those governments, who felt that it deserved the utmost encouragement. The work before us is another example in illustration of the above position; and its execution, aided, as the compiler has been, by men of science in his undertaking, is such, that we have no hesitation in pronouncing it the best production of its kind.

As no work of this description can be complete, without embracing the consideration of many medical and physical facts, we have deemed the volume before us the more worthy of our attention, as we find it to contain a vast variety of facts of that description, which it would be almost a dereliction of duty on our part to keep from the knowledge of our readers.

The avowed object of the present statistical inquiries, by the prefect of the department of Paris, we are informed in the preface, was the accurate enumeration of the population of that city; an operation that had hitherto been but imperfectly performed, owing to a number of obstacles, which have, only lately, been surmounted. The means resorted to for overcoming those obstacles are detailed, at full length, in the body of the work, and are distinguished for their novelty and ingenuity, as well as for the sagacity of their contriver. In carrying into effect an operation of such importance, it was scarcely possible to avoid entering into the investigation of several collateral objects of statistical knowledge. Hence we find, in the volume before us, a description of the territory and climate of the department,—that of the public establishments,—with an exact enumeration of the articles of food, industry, agriculture, and trade. But our principal reason for noticing this work is to give a summary account of the numerous tabular results it embraces, connected with many points of hygiene. These official documents are now printed for the first time; and it is to be hoped that the French government will permit their publication, and consequent circulation, throughout Europe; where they cannot fail to prove a source of emulation, and a model worthy of imitation, to other governments. We are happy to learn, from the concluding paragraph of the introduction, that the present is only the first of a series, which it is intended to publish, from time to time, under the same authority.

The work begins with some general notions on population, wherein the fundamental and theoretical principles, on which that question depends, are fully developed.

This first memoir is divided into four sections, and treats of the following subjects:—1. On the ultimate object of every inquiry into the state of population, and on the tables which represent the results of such inquiries. 2. On the use of public registers, for the purpose of constructing tables of mortality and population. 3. On the general properties of these tables. 4. General considerations on the causes which influence the laws of population.

Of the many curious facts contained in these four sections, we hold the most important to be that which relates to the probabilities of life, deduced from the annual bills of mortality, and the progressive diminution in the number of persons born in the course of the same year.

A table, founded upon these principles, is annexed to the memoir; by means of which, every problem, connected with the question of population, can easily be solved. With the assistance of this table, it will be a matter of no difficulty to determine—1, how many individuals are living at any one time, whose age surpasses any given number of years; 2, how many inhabitants there are whose age is comprised within two given periods; 3, what number of individuals, of a given age, die in the course of the year; 4, what is the mean duration of life, and what are the probabilities relative to the duration of life; 5, what are the chances of any man, of a given age, dying before he is a given number of years older; 6, what probability exists of two persons, of *different* ages, living an equal number of years longer.

These important problems, however, are not the only ones that can be solved by means of the table in question. Similar problems, relative to physical and medical questions, are here brought forward, and equally resolved by it; and a singular, but perfectly correct, deduction is obtained from all these data, on which, indeed, the value of every statistical inquiry depends,—viz. "que la repetition indefinie des evenemens que l'on regarde comme fortuits, faits disparaître tout ce qu'ils ont de variable."

The next paper of importance, contained in the volume before us, is an extract of the report made by the prefect to the minister of the home department; giving an account of the measures adopted for the purpose of accurately ascertaining the population of Paris. The reporter remarks, that the same operation had been undertaken on several occasions before; but that, owing to various circumstances, the results obtained had always proved inexact.

We are much mistaken if, in the course of the last census ordered by the House of Commons, a number of errors, such as have often been committed in France, in similar operations, and are now, for the first time, pointed out in the volume before us, have not crept in, and rendered the returns imperfect. It would be well for those who have the revision of those returns, to test them by the formulæ contained in the volume under consideration.

From the returns of the last census for the city of Paris, it appears that there were in that metropolis 720,000 inhabitants.

The numerous tables which accompany the two memoirs we have analyzed, and to which we have alluded, are classed under different heads:

A. Meteorology, 2 tables.

B. Population: *a*, census, 9 tables; *b*, *mouvement* of the population for 1817 and 1818, 24 tables; *c*, number of deaths from pulmonic diseases, in the course of the years 1816, 1817, 1818, and 1819, one table, founded upon the reports and declaration of the physicians and surgeons commissioned to ascertain the causes in every case of death.

C. Public hospitals, and other medical charities, 10 tables.

D. Agriculture, 4 tables.

E. Consumption of food, 5 tables.

F. Public instruction, 3 tables.

G. Fine arts, 2 tables.

H. Carriages and means of transport.

Of all these tables, those which must necessarily interest our readers are, the table of pulmonic diseases, and those relating to public hospitals and other medical charities. Even among those which regard the progress of population, there are some deserving of consideration. We shall endeavour to extract from them whatever results we think likely to be of service to our readers; and, where it can be done consistently with the nature of our Journal, we shall insert the table itself, in order to afford a greater degree of information.

As a specimen of these tabular arrangements and demonstrations, and as an object of juridical medicine, we beg leave to copy, for the present, the enumeration of suicides committed or attempted in the department of the Seine, during the year 1817. The other tables will follow in successive Numbers.

**Table of Suicides**, *committed or attempted, in the Department of the Seine, during the Year* 1817.

| Suicides | | |
|---|---|---|
| Followed by death, 285 | Total of Suicides, 351 | Not followed by death, 66 |

| Effected or attempted, | | | | |
|---|---|---|---|---|
| By men, 235 | By women, 116 | Total, 351 | By batchelors, 165 | By married persons, 186 |

| *Means of Destruction employed.* | *No. of Suicides.* | *Total.* | *No. of Suicides.* | *Presumed Motives for the Suicide.* |
|---|---|---|---|---|
| Voluntary falls .... | 39 | 351 | 22 | Love. |
| Strangulation....... | 36 | | 128 | Diseases, hypochondriasis, weakness, mental alienation, domestic quarrels, and grief. |
| Cutting or other instruments .. | 23 | | 15 | Bad conduct, gaming, lottery, debauchery, &c. |
| Fire-arms ........ | 46 | | 89 | Poverty, loss of places or employments, derangement of affairs. |
| Poison............. | 13 | | 15 | Dread of reproaches and punishments. |
| Suffocation by the vapour of charcoal .......... | 35 | | 52 | Motives unknown. |
| Do. by submersion | 160 | | | |

